# Histological features of *Jaculus jaculus* male-reproductive system: detection of metalloproteinases in testes and seminal vesicles during the active-reproductive phase

**DOI:** 10.1590/1984-3143-AR2025-0117

**Published:** 2026-07-24

**Authors:** Nesrine Zeghloul, Nemcha Lebaili-Benmoussa, Mohamed El Fadel Ousmaal, Salim Lamine, Nesrine Lina Gharnaout, Sabrina Lekmine, Nassim Moula, Walid Elfalleh, Fehmi Boufahja, Hamdi Bendif, Hichem Tahraoui, Abdeltif Amrane

**Affiliations:** 1 Department of Biological Sciences, Higher Normal School of Kouba Bachir El Ibrahimi, Algiers, Algeria; 2 Laboratory of Valorization and Bioengineering of Natural Resources – LVBRN, Faculty of Sciences, University of Algiers, Algiers, Algeria; 3 Higher School of Saharan Agriculture Adrar, Adrar, Algeria; 4 Department of Life and Environmental Sciences, University of Cagliari, Cagliari, Italy; 5 Biotechnology, Water, Environment and Health Laboratory, Abbes Laghrour University, Khenchela, Algeria; 6 Fundamental and Applied Research in Animal and Health – FARAH, Department of Veterinary Management of Animal Resources, Faculty of Veterinary Medicine, University of Liege, Liege, Belgium; 7 Department of Biology, College of Science, Imam Mohammad Ibn Saud Islamic University (IMSIU), Riyadh, 11623, Saudi Arabia; 8 Laboratory of Biomaterials and Transport Phenomena – LBMTP, University Yahia Fares, Medea, Algeria; 9 Ecole Nationale Supérieure de Chimie de Rennes, Université de Rennes, Rennes, France

**Keywords:** desert jerboa, reproductive cycle, MMP-2, MMP-9, phagocytic activity

## Abstract

Our study explored the histological and histo-functional characteristics of the testis and seminal vesicles of *Jaculus jaculus*, collected from the Biskra (34° 51' 0" N 5° 43' 59.999" E) and M’sila regions in Algeria (35°42'20.99" N 4°32'30.98" E) from autumn to early spring, corresponding to activity and transitional periods of the reproductive cycle. An immunohistochemical analysis of matrix metalloproteinases (MMP-2 and MMP-9) was conducted on the seminal vesicles and testes during the active reproductive. The study utilized an indirect immunohistochemistry protocol with amplification through streptavidin-biotin-peroxidase. Histologically, seminiferous tubules in *Jaculus jaculus*, captured during the transitional period of the reproductive cycle, multinucleated giant cells of germinal origin were observed during the transitional phase of the reproductive cycle, reflecting a process of seasonal testicular remodelling. The histological structure of seminal vesicles appears highly characteristic of the studied species, with the vesicular epithelium organized into complex acinar glands surrounded by basal lamina and connective tissue. Gland cells secrete a heterogeneous product. In the testis, cells of the seminiferous epithelium express MMP-2 and MMP-9 in their cytoplasm, with a strong expression of MMP-2; however, both proteins are absent from the extracellular matrix. During the active period, in seminal vesicles, a significant immunohistochemical signal of MMP-2 and MMP-9 was observed in epithelial cells, smooth muscle cells, with no immunoexpression in the extracellular matrix. MMP-2 and MMP-9 are expressed in a pattern consistent with a potential role in the seasonal reproductive cycle.

## Introduction

The sustainability of an animal species hinges on its capacity to adapt to environmental conditions and uphold reproductive activity (Kang et al., 2024). Environmental factors and nutritional status exert a significant influence on the reproductive cycle (Yang et al., 2025). Rodents, particularly those inhabiting temperate regions and arid or semi-arid areas, exhibit a seasonal reproduction strategy characterized by periodic shifts between activity and sexual rest. The adjustment of reproductive function is a result of synchronizing this activity with environmental conditions, manifesting in the modulation of births across different periods throughout the year (Amirat et al., 1980; Atabakhsh et al., 2018). *Jaculus jaculus*, commonly known as the "desert jerboa," is a small nocturnal rodent species, belonging to the Dipodidae family, ound in arid and semi-arid regions (Meunier et al., 2020). These gerbils are widely distributed in North Africa, including the Algerian sahara, the western region of Iran, and the Arabian Sahara. Its presence has been confirmed in various Algerian regions such as Doucen in the wilaya of Biskra, as well as in Batna, Djelfa, El Bayadh, and Tebessa. Only a handful of studies have focused solely on the reproductive strategy of *jaculus jaculus* with older studies, with animals captured from Sudan, revealing that breeding and pregnancy was observed during the winter period (Happold, 1967; Ghobrial and Hodieb, 1973).

Male reproductive function is regulated by androgens, which exert their effects by binding to nuclear androgen receptors (Guo et al., 2025a, b). These hormones regulate numerous functions and play a crucial role in pronounced seasonal fluctuations in weight, as well as in structural and ultrastructural changes of the reproductive system (Carson-Jurica et al., 1990; Yang et al., 2025). The cessation of reproductive activity is marked by structural and functional atrophy of reproductive organs, including testis and seminal vesicles, associated with reduction in hormone production (Khammar and Brudieux, 1987). Several studies have shown that interactions between pollutants and biological stressors such as viral infections can enhance reproductive toxicity (Hu et al., 2024; Zhang et al., 2025). Extensive research on desert rodents, such as Psamommys, Meriones, and Girbillus, has thoroughly explored the seasonal cycles of both endocrine and exocrine testicular function (Hegelen and Alizee, 2012). These structural seasonal variations indicate tissue remodeling, likely occurring through processes involving the degradation and re-synthesis of extracellular matrix proteins, facilitated by various factors such as plasminogen activators and proteases (MMPs) (Walsh et al., 2007).

Matrix Metalloproteinases (MMPs), a category of extracellular endopeptidases that depend on Zn^2+^ and Ca^2+^ for their function (Visse and Nagase, 2003; Yang et al., 2023), play a role in the remodeling of tissues associated with normal physiological processes, such as reproduction and embryonic development (Dubois et al., 2000; Page-McCaw et al., 2007). They possess the capability to degrade components of the extracellular matrix as well as non-matrix proteins, including growth factors, their receptors, cytokines, chemokines and adhesion proteins (Shahed and Young., 2008, Nakamura et al., 2005), this activity establishes a favorable microenvironment for processes like cell migration, cell differentiation, growth, proliferation, and apoptosis (Nagase et al., 2006; Rodgers et al., 2009). In a manner analogous to their impact on extracellular matrix proteins, MMPs can alter tissue structure (Hulboy et al., 1997; Stamenkovic, 2003). For rodents exhibiting seasonal reproductive activity, the sexual resting period is marked by structural atrophy in reproductive organs, reflecting a matrix metalloproteinases dependent tissue remodeling (MMPs) (Metayer et al., 2002). Indeed, MMPs play a pivotal role in the physiological variations of various organs within the male reproductive system and actively contribute to all transformations in the extracellular matrix (Walsh et al., 2007). The presence of MMPs significantly contributes to gamete fusion during fertilization and the acrosomal reaction, suggesting their involvement in sperm maturation within the testicular fluid of domestic animals (Aknoun-Sail et al., 2017). During spermatogenesis, germ cells must traverse the blood-testis barrier to move from the basal compartment to the luminal compartment in the testis (Metayer et al., 2002). MMP-2 and MMP-9 are detected in the testis and semen, and their enzymatic levels are associated with semen parameters (motility, concentration) in dogs, suggesting a functional involvement in sperm formation and quality (Warinrak et al., 2015). Although direct data in the testis are limited, studies on reproductive cells show that the expression of MMP-2 and MMP-9 is influenced by hormones (FSH) and growth factors, implying a potential interaction between hormonal pathways and MMP regulation in reproductive tissues (Portela et al., 2009). *In vitro*, some studies have highlighted the role of MMP-9 in remodeling the barrier between Sertoli and germ cells, particularly during the transition of germ cells across this barrier, indicating that these enzymes could modulate the permeability or restructuring of the barrier during spermatogenesis (Lydka et al., 2012). Importantly, normal spermatogenesis is regulated by the secretion of testicular as well as by the normal secretion of pituitary FSH and LH (Tang et al., 2020; Witzmann et al., 2003).

This study aimed to investigate the histological features of the male reproductive organs (testis and seminal vesicles) in the small jerboa *Jaculus jaculus*. Our objective was to document the histo-functional changes occurring during both the active phase and transitional period of the reproductive cycle. Additionally, we aimed to demonstrate the immunohistochemical presence of metalloproteinases (MMP-2 and MMP-9) in the testis and seminal vesicles of *Jaculus jaculus* throughout the active reproductive phase. The purpose of this investigation was to shed light on their potential role in the reproductive physiology and tissue remodeling processes affecting these glands during the seasonal reproduction. We hypothesized that the expression of MMP-2 and MMP-9 varies between the active and transitional phases of the reproductive cycle, reflecting their involvement in seasonal tissue remodeling.

## Methods

### Animals

Our study was conducted on 15 adult male of the desert jerboa *Jaculus jaculus*, and weighing between 57-73 g. Animals were captured from September 2023 to December 2024 in two semi-arid regions of the Algerian Sahara: LOUTAYA, located 25 km Northwest of Biskra province (35° 02´ 00ʺ N, 5° 36' 00ʺ) and the region of *Ain Lahdjel* of M'sila province (35° 40′ 26″ N, 3° 52′ 54″E):

- The first group was gathered in September in Loutaya (Biskra Province) and included six (06) adult males weighing between 57-69 g during the active period of reproductive phase;
- The second group was gathered in December in Loutaya (Biskra Province) and included seven (07) adult males weighing between 61-73 g during the active period of reproductive phase;
- The third group was gathered at the end of March in Ain-lahdjel (M'sila Province) and included three (03) adult males weighing between 57-68 g during the transitional period of the reproductive.

To minimize stress, animals were placed in separate cages provided with the plant from the biotope and maintained under the temperature and photoperiod conditions of the season for a maximum of 10 hours to prevent the influence of the nychthemeral rhythm. Under deep ketamine anesthesia (150 mg/kg, intraperitoneally), animals were euthanized, and tissue collection was conducted entirely within the laboratory under controlled conditions, with all samples collected at the same time of day to minimize potential circadian variations; the right testis was carefully excised and weighed.

All procedures were carried out in accordance with the guidelines of the Algerian Association of Experimental Animal Sciences (AASEA) and were approved by the local ethics committee of Houari Boumediene University of Science and Technology (USTHB), Algeria (Approval No. 45/DGLPAG/DVA.SDA.14). The laboratory work was conducted at the Laboratory of Animal Ecology within the National Superior School of Kouba in Algiers.

### Anatomo-structural stady of the testis and seminal vesicles

The testes and seminal vesicles underwent dehydration using a graded series of ethanol, followed by clearing in cyclohexane and embedding in paraffin. Serial sections of 4 µm thickness were then prepared for staining.

### Immunohistochemical study of MMPs (MMP-2 and MMP-9)

This study employed an indirect immunohistochemical method using streptavidin-biotin-peroxidase amplification following the KIT LSAB2 (DAKO) peroxidase immunohistochemistry protocol, as outlined by MacNeil et al. (2020): Histological sections (4 µm thick) were mounted on silanized slides, deparaffinized in xylene, and rehydrated through a graded series of alcohols, followed by rinsing in distilled water. Heat-induced antigen retrieval was performed at 95 °C in a water bath using a Tris–EDTA buffer (pH 6 or pH 9) containing 0.05% Tween-20. The sections were then washed in Tris-buffered saline (TBS), and endogenous peroxidase activity was blocked by incubation in 3% hydrogen peroxide (H_2_O_2_) for 10 minutes. The sections were incubated with primary antibodies as follows: The primary antibody against -MMP-2 (mouse monoclonal anti-human; Invitrogen; dilution 1:70; pH 6; clone 101; unconjugated; Cat. #436000) was applied for 1 hour; The primary antibody against MMP-9 (mouse monoclonal anti-human; Invitrogen; dilution 1:100; pH 9; clone IIA5; unconjugated; Cat. #MA5-14228) was applied to separate sections for 1 hour. Following incubation, a secondary antibody conjugated to the streptavidin–biotin–peroxidase complex was applied. Immunohistochemical visualization was carried out using 3,3′-diaminobenzidine (DAB) chromogen, followed by counterstaining with hematoxylin. Finally, the sections were dehydrated, mounted, and examined under a light microscope.

### Staining techniques

In this study, specific histological staining methods were employed, including Periodic Acid–Schiff (PAS) staining, Masson’s Trichrome staining, and Azan Trichrome staining.

### Statistical analysis

To determine significant differences between the two groups, animals in the active period of the reproductive phase and animals in the transitional period of the reproductive phase, the normality of variable distributions was first assessed using the Kolmogorov-Smirnov test. Differences between groups were then analyzed using a two-tailed Student’s T-test and considered statistically significant at p < 0.05. No data points were excluded during the analysis. All values are presented as means ± standard error. Statistical analyses were performed using GraphPAD InStat (Prism 9.0 package, San Diego, CA).

## Results

Our study is organized into two main parts. The first includes the results of the anatomo-structural and histo-functional analysis of the gonads and seminal vesicles to determine the modifications specific to the active phases of the reproductive cycle of *Jaculus jaculus*. The findings derived from the histological and immunohistological examinations of the testicular and seminal vesicular tissues of *Jaculus jaculus* during both sexual activity and the transitional phase of the reproductive cycle are notably intriguing. Meanwhile, the second part includes identification and distribution of metalloproteinase (MMP-2 and MMP-9) along the reproductive phase.

**Figure 1:**
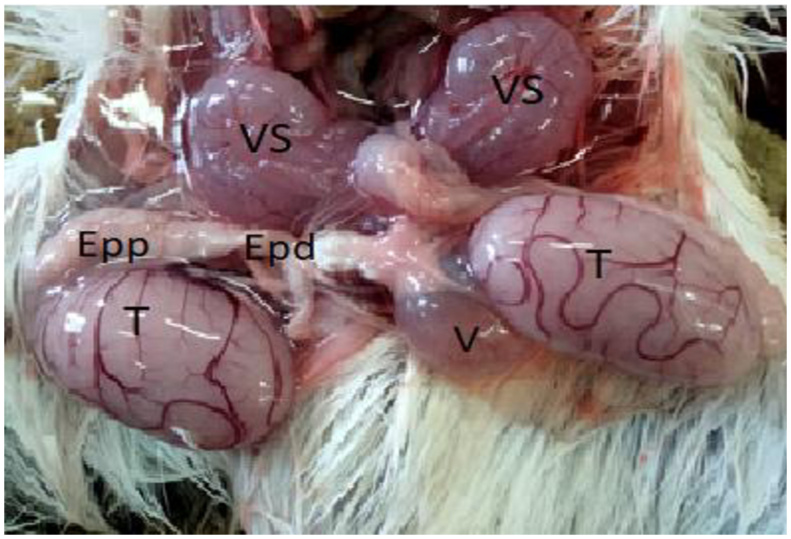
Reproductive anatomy of adult *Jaculus jaculus* in the active period of reproductive cycle, testis (T), seminal vesicles (VS), proximal Epididymides (Epp) and distal Epididymides (Eed).

The male reproductive system of *Jaculus jaculus* closely resembles that observed in other rodent species, encompassing the testis, excretory ducts, appendix glands, and penis, as illustrated in Figure 1.

### Anatomo-histological characteristics of the testes in *Jaculus jaculus*

During the phase of sexual activity, the reproductive system displays well-developed testes, measuring approximately 3.5 cm in length and around 1.5 cm in width. These testes exhibit a smooth surface appearance and significant vascularization, appearing whitish. The studied rodents exhibited a body weight ranging from 59 to 73 g (**Table 1**), while testicular weight varied from 2.4 to 2.8 g. Relative testicular weight evaluations indicate that the testes account for between 3.3% and 4.8% of the body mass, reflecting a proportional increase in gonadal size during the sexual activity period.

**Table 1:** Comparison of body weight, absolute testicular weight, and relative testicular weight between the active reproductive period and the transitional period.

**Parameter**	**Active period**	**Transitional period**	***P* value**
Body weight (g)	64.52±1.47	62.3±3.18	NS
Absolute testicular weight (g)	2.64±0.03	1.713±0.03	****
Relative testicular weight (g)	4.10±0.05	2.759±0.08	****

Values are given as means ± SEM. Significant differences were analyzed using Student test and indicated by ****P < 0.0001. NS = not significant.

In the transition phase, partial regression of both body weight (57-68 g) and a significant decrease of absolute and relative testicular weight (1.66-1.78 g; 2.4-3.1% of body weight) is observed compared to the active phase. This reflects preparation for the sexual rest phase, corresponding to a reduction in gonadal activity, typical of seasonal adaptations in desert-dwelling rodents. This phenomenon represents energy redistribution, where investment in reproduction is temporarily reduced in favor of maintaining vital functions in an often challenging environment.

In *Jaculus jaculus*, the gonadosomatic index in adult males ranges from 6.6% to 9.8%, reflecting a high reproductive investment and strong spermatogenetic activity. These values indicate high sperm activity and significant sperm production, characteristic of a species with an intense reproductive strategy during the active phase. In desert gerbils such as *Gerbillus tarabuli*, both indices and dimensions vary greatly between the active and quiescent periods, suggesting an adaptation to environmental seasonality, with a GSI ranging from 0.36% to 0.68% (Hamidatou Khati and Hammouche, 2021). Studies on *Gerbillus gerbillus* and *Psammomys obesus* also showed clear seasonal testicular weight changes indicative of fluctuating GSI through the reproductive cycle (Boufermes et al., 2014).

Examining the sections allowed us to delineate the histological features and distinctive cellular components characteristic of the species and the specific reproductive cycle period. In a cross-section, the testis unveils numerous seminiferous tubules, featuring lumens abundant in spermatozoa compared to those appearing vacant. Each seminiferous tube is enveloped by a thin outer membrane containing contractile cells along with a stratified and well-developed germinal epithelium, possessing a thickness equivalent to two-thirds (2/3) of the tube's radius.

**Figure 2:**
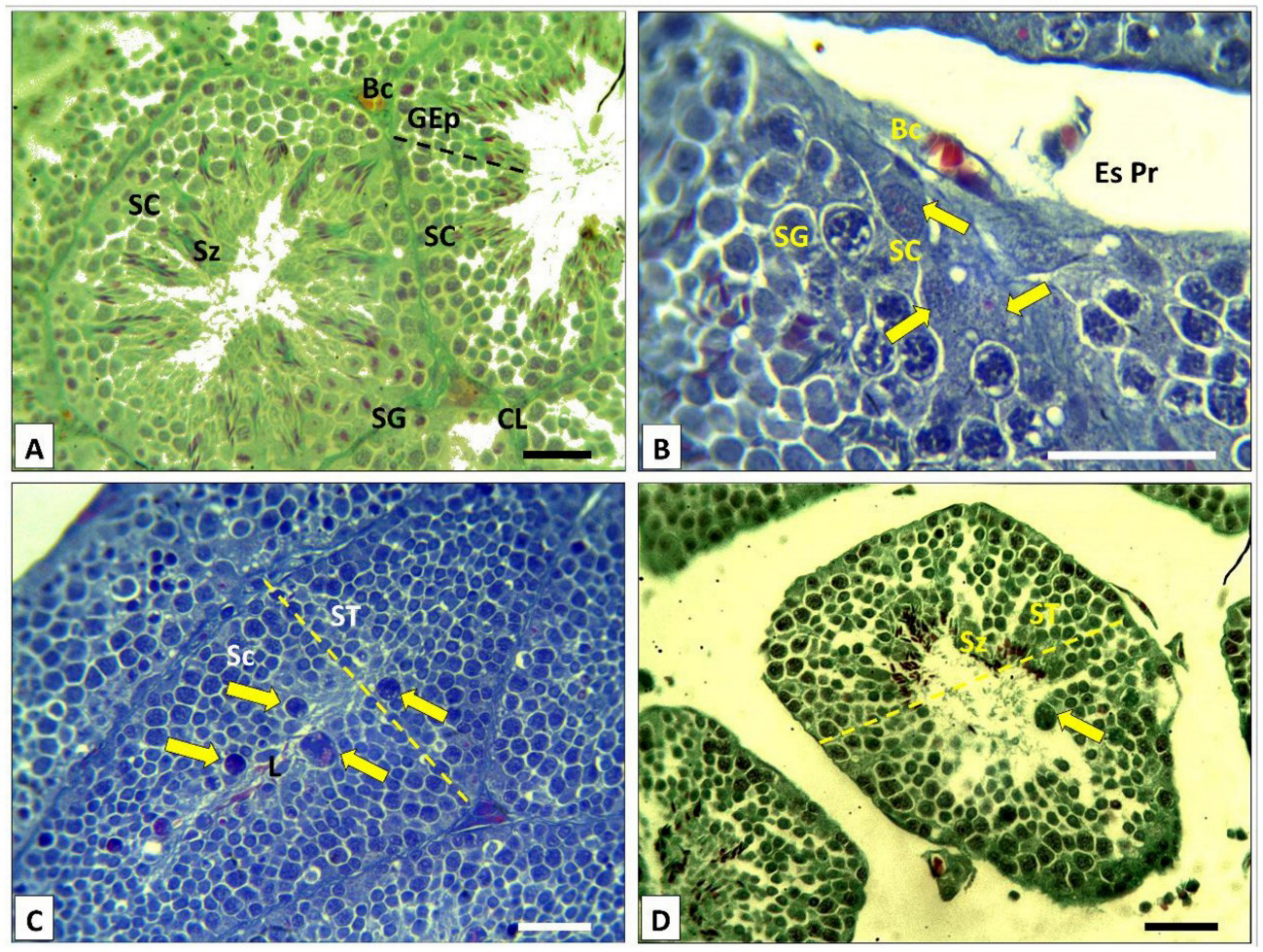
Histological characteristics of the seminiferous tubules in adult *Jaculus jaculus* in the active period. **A, B** Cross-sections through seminiferous tubules (ST) showing that germinal epithelium (GEp) present Spermatozoïds (Sz), Spermatocyts I (Sc), Spermatogonia (SG) as well as Sertoli cells (SC) poly-nucleic (arrows). Peritubular space (EsPr) rich in blood capillaries (Bc) and Leidig cells (CL). **A** Azan staining Bar: 20 µm; **B** Masson's Trichrome staining Bar: 20 µm. **C** and **D** presence of particular cells in the germinal epithelium (arrows). **C** Masson's Trichrome staining Bar: 20 µm; **D** Azan staining Bar: 20 µm.

At higher magnifications, the seminiferous epithelium revealed distinct cell populations. The basal layer of the epithelium included undifferentiated germ cells known as spermatogonia, Sertoli cells, and differentiating germ cells. The somatic Sertoli cells, positioned adjacent to and interspersed among the germ cells, extend throughout the thickness of the germinal epithelium. These cells exhibit an elongated pyramidal shape, and the boundaries of their cytoplasm are less discernible due to their close contact with the germ cells. Their large nuclei with clear chromatin and one or more prominent nucleoli is frequently situated on the basal side (as illustrated in Figure 2B).

The histological examination of the seminiferous epithelium revealed the presence of certain cells with distinctive characteristics, including a polylobed nucleus and a granular cytoplasm and is situated within the tubular lumens. In seasonally reproducing species, testicular regression is associated with disruption of the cell junctions within the seminiferous epithelium and a reduction in the integrity of the blood–testis barrier, a phenomenon reported in *Talpa occidentalis* and other seasonal mammals (Dadhich et al., 2013; Jiménez et al., 2015).

However, in *Jaculus jaculus* during the transitional period of the reproductive cycle, the presence of cells compatible with immune cells in the lumen may reflect a temporary alteration of this barrier. This situation is likely associated with structural remodeling of the seminiferous epithelium, germ cell degeneration, and the resorption of germ cells, a phenomenon described in seasonally reproducing species (Massoud et al., 2018; Young and Nelson, 2001) (as depicted in Figure 2C).

**Figure 3:**
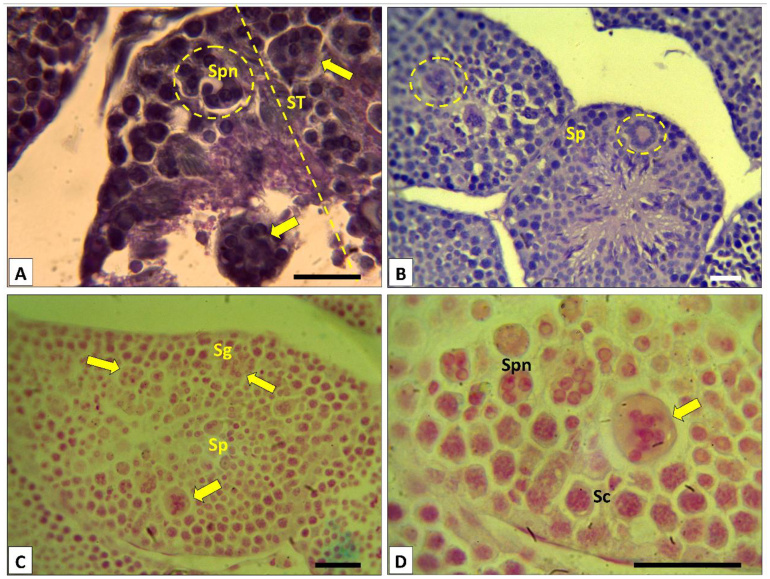
Histological features of seminiferous tubules in adult *Jaculus jaculus* in the transitional phase of the reproductive cycle. **A** during the transitional period of the reproductive cycle, cross-section through seminiferous epithelium (ST) shows giants cell containing numerous nuclei and homogeneous cytoplasm (arrows), contribute to reducing the number of spermatids during the transition from the active phase to the sexual rest phase. Masson's Trichrome staining. Bar: 20 µm. **B, C** and **D** polynuclear structures associated with spermatid degeneration. **B** Masson's Trichrome staining. Bar: 20 µm; **C** and **D** PAS staining. Bar: 20 µm.

In histological sections captured during the transitional stage of the reproductive cycle (Figure 3B), the seminiferous tubules of *Jaculus jaculus* exhibited noteworthy features. Particularly in the testes of *Jaculus jaculus* collected in March, a remarkable cellular structure emerged. This structure manifested as well-defined, closed, round or oval cell with significant volume, housing numerous peripheral spermatid nuclei and a homogeneous cytoplasm (Figure 33D). At higher magnification, closer examination suggests that these cells likely result from processes of spermatid degeneration and/or phagocytosis (Figure 3A).

### Histo-functional organization of the seminal vesicles in *Jaculus jaculus*

Under low magnification, an extended section of the seminal vesicle in the small jerboa during sexual activity revealed its composition. The seminal vesicle exhibited a robust casing encompassing a muscular layer organized into two distinct transverse and longitudinal muscular tunics. The mucosa, influenced by an epithelial layer and vascular connective tissue, displayed a configuration with numerous irregularly anastomosed diverticula, forming primary and secondary folds within the vesicle lumen (as shown in Figure 44B). This structural organization facilitates an increased surface area for exchange.

**Figure 4:**
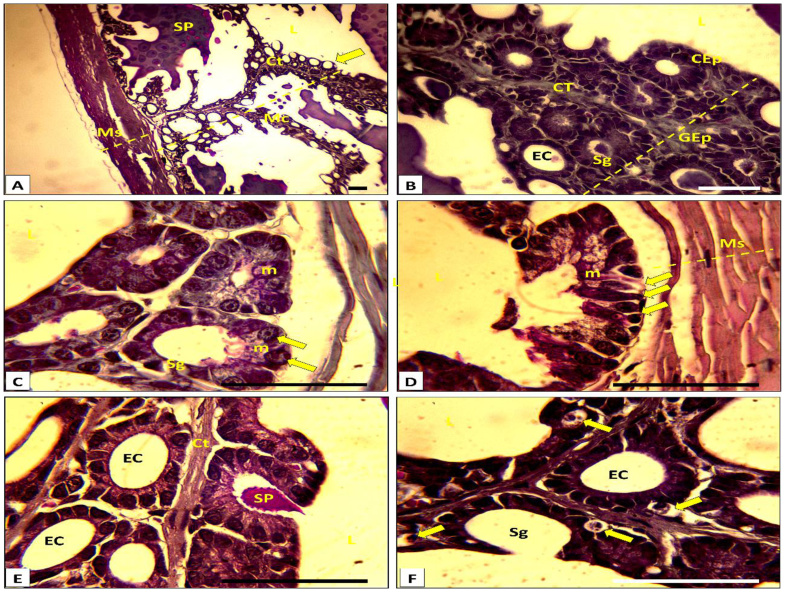
Histological characteristics of seminal vesicles in adult *Jaculus jaculus* during active period. **A, B** and **C** Cross-section in seminal vesicles shows: Muscular (Ms) with two layers of smooth and contractile muscle cells transverse and longitudinal, Mucosa (Mc) composed of connective tissue (Ct) and coating epithelium (CEp). Epithelial cells diversify their secretion products (SP) in the lumen (L). The vesicular epithelium (GEp) is organized in acinar secretory (Sg) exocrine and excretory canal (EC). The product of secretion is mucosal in nature (m). Masson's Trichrome staining. Bar: 40 µm. **D, E** and **F** secretory acinus (Sg) structure showing two types of cells (arrows) secreting into the lumen of the organ (L) two types of immiscible products (**D**). **F** shows the presence of a particular cell associated with the secretory part and bathes in the connective tissue (arrows). **D, E** and **F** Masson's Trichrome staining. Bar: 40 µm.

### Particularities and histological classification of the epithelium of the seminal vesicle in *Jaculus jaculus*

The seminal vesicle histology of the jerboa unveils a distinctive organization in the structure of its vesicular epithelium, which is reported here for the first time and appears to be unique to the species. Under high magnification, the epithelium exhibited a configuration comprising both simple and intricate secretory acini (rounded appearance with a reduced cavity), encircled by a basal lamina and connective tissue (as depicted in Figure 4C). The polarized secretory cells within the gland exhibited two distinct variations. The first type consisted of tall cylindrical cells with a ciliated apical pole (striated plate), featuring a basal, rounded, voluminous nucleus with clear chromatin occupying 1/3 of the cell volume, along with granular and clear cytoplasm (as seen in Figure 4C). A detailed observation of the apical pole of these cells under high magnification revealed that the mucosal nature of the secretion product was discharged into the vesicle lumen through the apocrine mode, with the product being released en bloc (Figure 4D). The second type involved elongated cells with an open apical pole, characterized by an elongated central nucleus and a dense cytoplasm.

Upon examining the histological sections, distinctive cells with spherical or ovoid shapes were evident. These small, compartment-enclosed cells were situated in the basal part of the acinus, closely positioned to blood capillaries. Characterized by a dense nucleus, granular cytoplasm, and an irregular outline, these cells appear to serve an immune or endocrine function, as illustrated in (Figure 4E). In cross-section, the excretory ducts, varying in diameter, exhibited a shorter length in smaller glands and a longer, branched structure in larger glands. The epithelium appeared as a simple cubic layer initially, gradually thickening and transitioning into a simple cylindrical and then stratified arrangement, as depicted in (Figure 4F).

### Immunohistochemical of MMP-2 and MMP-9 in the testes of *Jaculus jaculus* during the active period of reproductive cycle

**Figure 5:**
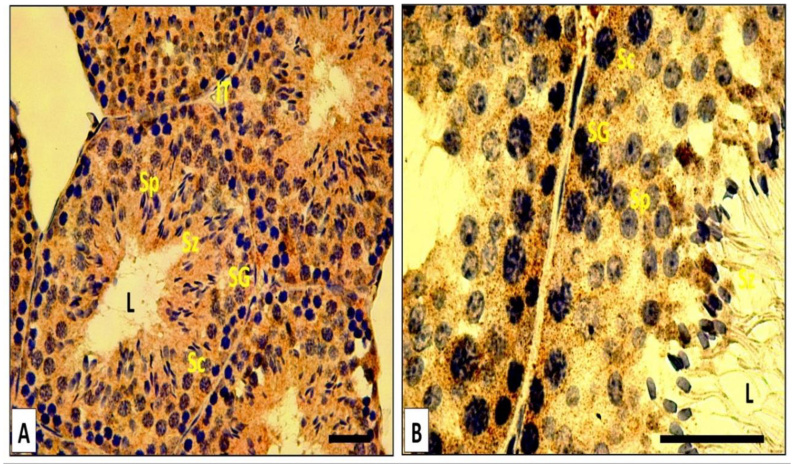
MMP-2 and MMP-9 immunohistochemistry in the adult *Jaculus jaculus* testis in the active period. **A, B** in the seminiferous tubule, all cells of the epithelium germinal: Spermatocyts (Sc), Spermatogonia (SG); Spermatids (Sp) Spermatozoids (Sz) express MMP-2 and MMP-9 in the cytoplasm. The immunostaining of MMP-9 is lower than that of MMP-2. L: Lumen. Bar: 20 µm.

During the active period in the testes, MMP-2 and MMP-9 exhibited high expression levels in various epithelial cells, including Sertoli cells, spermatocytes I, spermatocytes II, round and elongated spermatids (as depicted in Figure 55B). Particularly, the epithelial cells displayed a prominent immunohistochemical signal of MMP-2 (Figure 5A). However, the signal was notably low in the seminiferous lumen. Importantly, neither of these two proteins was detected in the extracellular matrix (ECM) that bounds the seminiferous tubules. In all negative controls, where the primary antibody was omitted, the immunohistochemical signal for both MMPs remained negative.

### Immunohistochemical of MMP-2 and MMP-9 in the seminal vesicles of *Jaculus jaculus* during the active period of reproductive cycle

**Figure 6:**
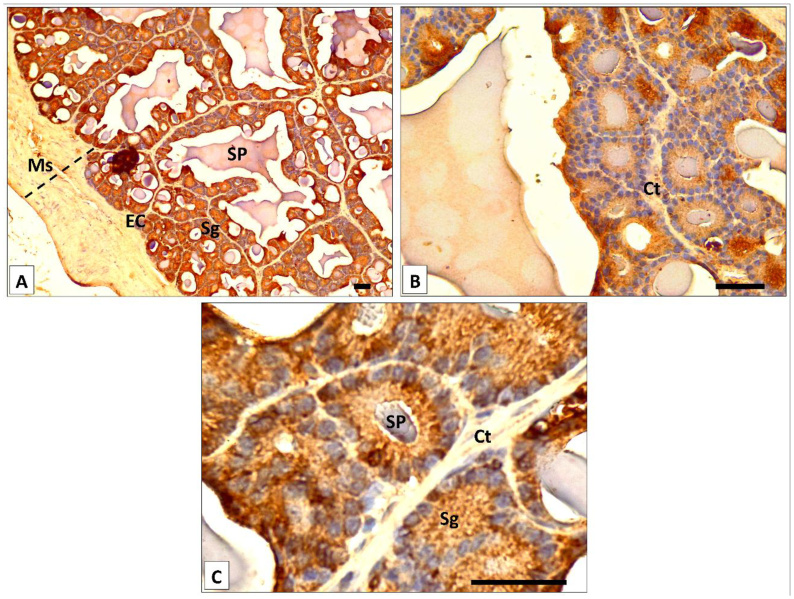
MMP-2 immunohistochemistry in the adult *Jaculus jaculus* seminal vesicles in active period. **A, B** and **C** Epithelial cells of the secretory glands (Sg) are too strongly immunolabelled, the connective tissue (Ct) is devoid of immunoreactivity. Smooth muscle cells (Ms) and secretion product (SP) show a significant immunoreactivity. Bar: 20 µm.

**Figure 7:**
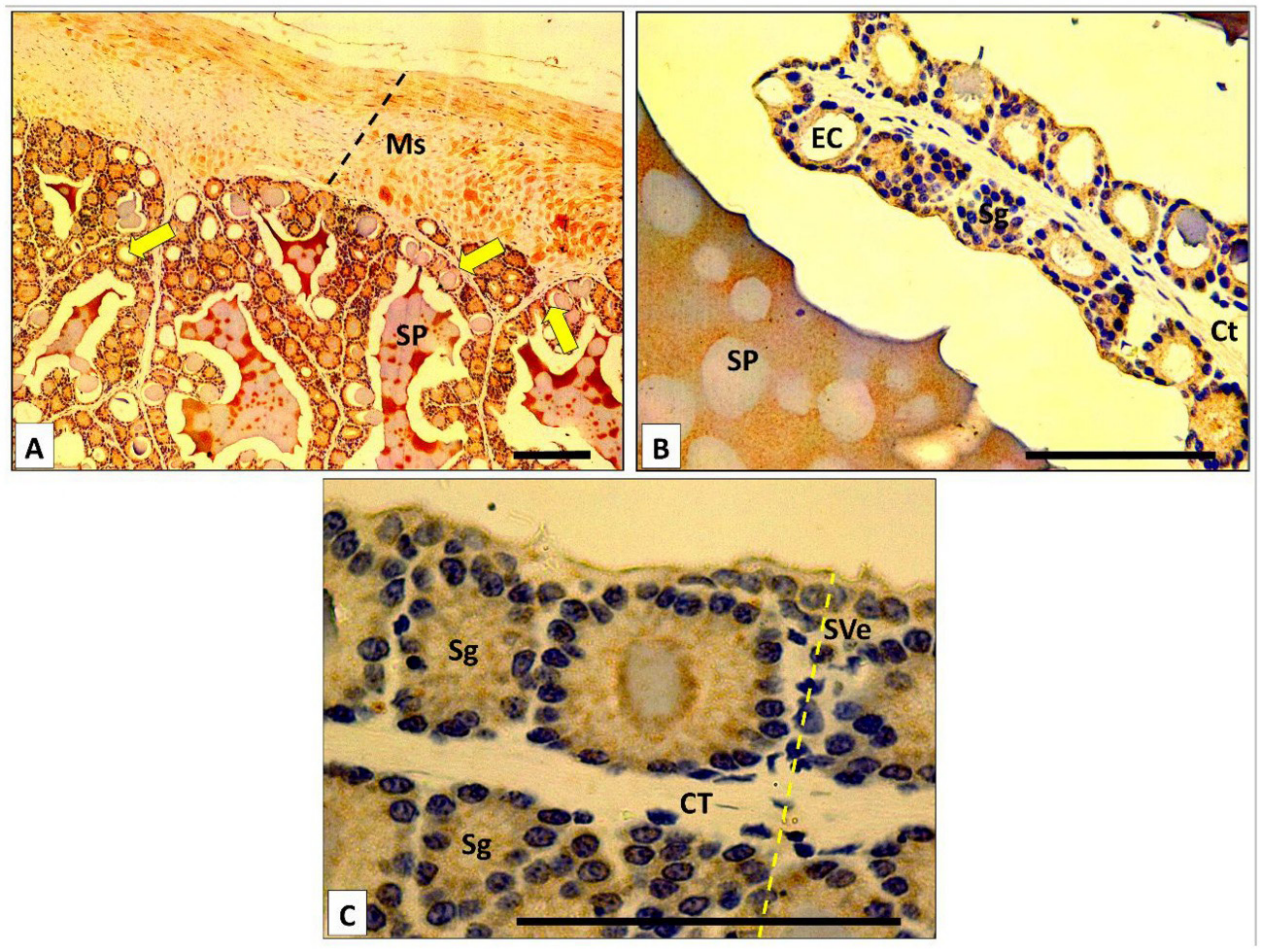
MMP-9 immunohistochemistry in the adult *Jaculus jaculus* seminal vesicles in active period. **A:** The immunohistochemical signal (arrows) was observed in epithelial cells; secretion (SP), the extracellular matrix and in the smooth muscle cells (Ms). In **B, C:** Epithelial cells of acini (Sg) and excretory ducts expressed a similar important immunostaining of MMP-9, and in the connective (Ct) tissue immunostaining is absent. SVe: segment of the seminal vesicular. Bar: 60 µm.

Within the seminal vesicles of *Jaculus jaculus*, MMP-2 immunostaining was evident in the cytoplasm of epithelial cells (acini and ducts) (illustrated in Figure 6A), smooth muscle cells (SMC), and the extracellular matrix (ECM). Notably, MMP-2 immunoreactivity was pronounced in epithelial cells and secretion, with a comparatively lower intensity in the SMC (Figure 66C). The connective tissue within the axis of the epithelial folds showed no MMP-2 immunolabeling (Figure 6C). For MMP-9 immunoexpression, it was observed in epithelial cells, SMC, and secretion products, mirroring the pattern observed for MMP-2. During the active period, both epithelial cells and SMC exhibited strong MMP-9 expression with equal intensity (Figure 77B). The ECM did not exhibit any signaling (Figure 7C), while the secretion's product appeared to be rich in MMP-9.

## Discussion

The synchronization of reproductive activities and metabolism is crucial, especially for species residing in challenging environmental conditions. *Jaculus jaculus*, a diminutive nocturnal rodent found in both temperate regions and arid or semi-arid areas, employs adaptation mechanisms primarily linked to reproductive function. This species demonstrates the capability to modulate its reproductive activity in accordance with seasonal changes.

### The histological particularities of testes and seminal vesicles in *Jaculus jaculus* during the seasonal reproductive cycle

The testes of *Jaculus jaculus* exhibit notably larger size and volume during the period of sexual activity when compared to other desert rodents such as *Gerbillus gerbillus* (Aknoun-Sail et al., 2017), *Meriones crassus* (Smai-Hamdidouche et al., 2015), *Meriones libycus* and *Meriones shawi* (Zaime et al., 1992). These studies indicate that nocturnal rodent species generally have more substantial testes than their diurnal counterparts, as evidenced by relative statistics and testicular weight. In *Jaculus jaculus*, the testes exhibit a distinctive morphology, resembling smooth beans on the surface and being relatively large, in contrast to the oval and very small testes observed in rats, hamsters, and gerbils.

In the transitional period of the reproductive cycle, histological sections of the small jerboa's testes reveal the presence of distinct, multinucleated giant cells characterized by numerous spermatid nuclei and a homogeneous cytoplasm are associated with massive spermatid degeneration, possibly of macrophagic origin. Their appearance coincides with seasonal testicular regression, which is characterized by extensive germ cell apoptosis and the accumulation of cellular debris. These cells are likely involved in phagocytic activity and testicular remodeling processes that facilitate the transition from the sexually active state to sexual quiescence. Confirmation of the histological nature of these cells requires immunohistochemical analyses (e.g., CD68, CD163) to distinguish a macrophagic origin from other cellular types. Similar phenomena have been reported in cases of testicular atrophy and tubular degeneration in humans and mice, as well as in rabbits (Holstein and Eckmann, 1986; Furukawa et al., 2023; Assar et al., 2023).

The development or regression of seminal vesicles, which are accessory glands, is androgen-dependent (Mansouria et al., 2011). In seasonally breeding rodents such as Mériones (Amirat et al., 1977), Psammomys and Gerbillus (Boufermes et al., 2021), significant histological modifications are observed in the seminal vesicles during the period of sexual activity. These modifications involve the three histological components, namely the epithelial part, mesenchymal fraction, and muscle fiber zones. In species like Mériones, Psammomys, and Gerbillus, the organ reaches its maximum development during sexual activity, presenting as anastomosed diverticula in the form of folds within the vesicle lumen. Each fold is bounded by a simple and glandular epithelium (Belhocine et al., 2015). Contrastingly, in *Jaculus jaculus*, the vesicular epithelium undergoes a complete transformation into exocrine glandular tissue with a complex secretory acinus. This unique organization, described for the first time, appears to be specific to the species. Notably, this epithelium has been reported as non-glandular in other desert rodents. Furthermore, the secretion product of the seminal vesicle in *Jaculus jaculus* appears heterogeneous and mixed, comprising two types of chemically immiscible molecules. This contrasts with other species of desert rodents where the secretion product of glandular cells appears simple and homogeneous.

### Identification of MMP-2 and MMP-9 expressions in the testes and seminal vesicles in *Jaculus jaculus* during sexual activity of reproductive cycle

The immunohistochemistry analysis revealed the presence of two matrix metalloproteinases (MMP-2 and MMP-9) in the testes and seminal vesicles of the desert jerboa (*Jaculus jaculus*) throughout the active period (Autumn and Winter) of the reproductive cycle. These MMPs were identified in epithelial cells, smooth muscle cells (SMC) of seminal vesicles. Previous studies have similarly detected MMP-2 and MMP-9 in the same cells in other species of desert rodents, such as Mériones and Psammomys (Kareskoski et al., 2021). This finding suggests that these enzymes play a role in intracellular processes, including synthesis and secretion, as well as in cell migration and differentiation. The SMC are believed to contribute to contractile activity, facilitating the expulsion of secretion products and participating in the tissue remodeling characteristics of the reproductive cycle period. These observations align with results from other rodent studies, such as *Meriones libycus* and *Psammomys obesus* (Legrand et al., 1999).

The detection of MMP-2 and MMP-9 in the seminal vesicle secretion indicates their intended export, providing additional evidence of their presence in seminal fluid (Buisson et al., 1996). Additionally, their involvement in extracellular processes and fertility is affirmed (Atabakhsh et al., 2018). These enzymes are likely to blend with seminal plasma, contributing to the maturation of spermatozoa. Notably, MMPs, being proteolytic enzymes, target both matrix and non-matrix substrates. However, maintaining an appropriate cellular concentration of MMPs is crucial for facilitating polarized cell migration (Rodgers et al., 2009).

In sand rats, examination of seminal vesicle protein homogenate during the sexual rest period revealed the activity of calcium-dependent metalloproteinases, as detected using the gelatin gel zymography method. Metalloproteinases play a crucial role in the reproductive organ development and spermatogenesis of adult rats (Metayer et al., 2002). They are also involved in re-epithelialization, with MMP-9 produced by epithelial cells, and regulation of cell migration (Ayvazova et al., 2016). Additionally, MMPs are generated by epithelial cells during healing, while stromal fibroblasts are the primary source of MMPs during cell invasion (Shahed and Young, 2008). During sexual activity, immunoreactivity of MMPs in the extracellular matrix of seminal vesicles is notably present which reflects a state of intense tissue remodeling. Indeed, during the active reproductive phase, gonadal tissues undergo significant structural modifications associated with hormonal stimulation, particularly testosterone, which promotes cell growth, differentiation, and reorganization of the extracellular matrix (ECM). These processes enable the tissue plasticity required for optimal reproductive function (Robinson et al., 2001). However, it should be considered that immunohistochemistry mainly allows tissue localization of protein expression and does not directly reflect the enzymatic activity of MMPs, which may be regulated by the presence of specific inhibitors such as tissue inhibitors of metalloproteinases (TIMPs). Therefore, although the intense immunostaining observed suggests a strong involvement of MMPs in ECM remodeling during the active reproductive period, complementary analyses—such as gelatin zymography and Western blotting—would be necessary to further elucidate the functional dynamics of these enzymes.

In the male reproductive tract organs studied, the connective stroma showed no immune response, indicating limited transcriptional or synthetic capacity. In our study, MMP-2 expression appeared higher than that of MMP-9 based on qualitative assessment. It is reported that MP-9 expression is generally weak or absent in normal tissues but can be induced in the context of tissue remodeling (Ayvazova et al., 2016).

In desert-dwelling rodents, sample size is often structurally limited, reflecting ecological constraints inherent to desert environments, including short seasonal and reproductive activity periods, high mortality associated with extreme heat and aridity, low population density, and pronounced spatio-temporal variability of the studied rodent populations (Pizzutto et al., 2021).

In addition, sample size was deliberately restricted to minimize the impact of sampling on naturally vulnerable populations, in accordance with ethical guidelines for wildlife research. The unequal distribution among experimental groups can be attributed to the natural variability in the reproductive status of individuals at the time of sampling, a phenomenon well documented in desert rodents with seasonal reproductive cycles (Pizzutto et al., 2021). Despite these limitations, the sample size was sufficient to reveal clear and biologically coherent reproductive trends, providing original data on a species that remains poorly studied. Collaborative studies conducted across multiple geographic sites would allow a substantial increase in sample size without over-sampling a single population, while accounting for the ecological variability characteristic of desert environments. The establishment of inter-site research networks, encompassing multiple populations distributed along an environmental gradient, would represent a relevant approach to strengthen the statistical robustness of the results. Furthermore, the use of non-invasive methods and longitudinal monitoring would offer promising perspectives for advancing the study of reproduction in desert-dwelling rodents, while respecting the ethical and ecological constraints specific to these species.

## Conclusion

In *Jaculus jaculus*, the reproductive season manifests distinct characteristics in histological organization of male reproductive organs, specifically the testes and seminal vesicles. Notably, the seminal vesicles undergo a significant transformation, with the secretory epithelium evolving into complex acinar glands differentiating two distinct types of glandular cells. Which result in the production of a heterogeneous seminal product. During the transitional period of the reproductive cycle, the testes exhibit polymorphonuclear cells, a phenomenon attributed to testicular remodeling that facilitates the transition toward sexual quiescence. This activity reduces the number of spermatids, marking the transition from the active reproductive phase to the period of sexual rest. Throughout the seasonal reproductive cycle of *Jaculus jaculus*, both the seminal vesicles and testes demonstrate the production of metalloproteinases (MMP-2 and MMP-9). These enzymes are expressed in patterns suggesting potential roles in various cellular activities, including the contractility of circular muscle layers (CMLs) to facilitate the discharge of secretion into the external environment. These findings significantly enhance our understanding of the intricate histophysiology of the reproductive system in semi-desert rodents.

## Data Availability

Data will be made available on request.
